# At the tipping point: nursing judgement, workforce sustainability, and patient safety

**DOI:** 10.1177/17449871261419019

**Published:** 2026-02-19

**Authors:** Sarah Bekaert

**Affiliations:** Oxford Brookes University, UK

The International Council of Nurses (ICN) has repeatedly warned that global health systems are approaching a tipping point, driven by workforce shortages, unsafe working conditions, rising violence against nurses and increasing moral and cognitive demands on practice (ICN, 2023, 2024). The ICN states that safeguarding nursing is therefore not only a workforce imperative but also a prerequisite for quality care and health system resilience worldwide. Recent international reporting reinforces this assessment, highlighting both the fragility of healthcare systems and the centrality of nurses within them, from attacks on healthcare facilities in conflict settings to accelerating global nurse migration driven by poor working conditions (ICN, 2023; WHO, 2025). Furthermore, these pressures are not confined to extraordinary circumstances. They are felt daily in wards, homes, and communities, where nurses are expected to deliver safe, compassionate, and evidence-based care amid rising complexity and constrained resources.

Situated within this global context, this issue brings together research, reviews, and perspectives that examine how nursing practice sustains healthcare at its most vulnerable points. Collectively the papers reveal a shared concern: how to sustain nursing’s capacity to protect safety, support decision-making, and exercise clinical judgement when the conditions for reflection, judgement, and care are increasingly under strain (WHO, 2025).

## Nursing-led interventions as foundations of safety and quality

Several contributions in this issue demonstrate that nursing-led interventions remain among the most effective, and often undervalued, mechanisms for improving patient outcomes. Structured education for families, fall prevention strategies (Batiha et al., 2025; Bekaert, 2025a), infection control practices (Fletcher et al., 2025), and transitional care programmes (Bekaert, 2025b; Garmy, 2025; Keklik et al., 2025; Mozzarelli et al., 2025) all illustrate how nursing translates evidence into everyday safety.

Early, planned education for mothers of infants with hydrocephalus offers a clear example. By equipping caregivers with knowledge, skills and confidence, nurse-led education reduces anxiety and caregiver burden while also lowering clinically significant complications such as shunt infections (Keklik et al., 2025). This reinforces a long-standing nursing insight: safety does not end at discharge, and education is not an adjunct but a core clinical intervention (Bekaert, 2025b).

Similarly, fall prevention research in this issue highlights the relationship between nurses’ knowledge and confidence, and patient outcomes. Although individual competence matters, these findings also expose a recurring challenge in patient safety discourse: nurses may know what to do, yet remain constrained by organisational conditions that limit their ability to act (Batiha et al., 2025; Bekaert, 2025a). Safety, therefore, emerges not only from training but also from systems that align staffing, workflow, and support with clinical realities (ICN, 2024).

Questions of safety and standardisation also arise in the review of catheter-related bloodstream infections in patients receiving parenteral nutrition. Here, long-held assumptions about sterile technique are challenged by a weak evidence base, shifting attention towards broader issues of education, consistency, and system-wide standardisation (Fletcher et al., 2025).

Transitional care for people with peripheral arterial disease extends this safety lens beyond hospital walls. Nurse-led transitional care programmes demonstrate how continuity, education, and follow-up reduce readmissions and improve quality of life (Garmy, 2025; Mozzarelli et al., 2025). In an era of shortened hospital stays and rising multimorbidity, continuity itself becomes a patient safety intervention (WHO, 2025).

## Relational care and shared decision-making in complex lives

Beyond technical safety, several papers foreground nursing’s relational and interpretive work, specifically in contexts where decisions are value-laden, emotionally charged, and embedded in everyday life. Shared decision-making with older adults receiving home care illustrates this complexity (Doan et al., 2025). Decisions about remaining at home or transitioning to other living arrangements are rarely purely clinical. They involve identity, family relationships, risk tolerance, and social resources. Nurses in home care settings report moderate-to-high involvement in such decisions, yet often feel less influential than family caregivers (Patel, 2025).

This theme resonates with studies on family education and transitional care. Whether supporting parents of infants with complex needs or adults recovering from vascular surgery, nurses operate as interpreters – helping patients and families understand risks, recognise warning signs, and align care with what matters to them (Bekaert, 2025a; Garmy, 2025).

Taken together, these papers suggest that shared decision-making is not a discrete skill but a practice environment. It requires time, training, organisational endorsement, and recognition that nursing knowledge includes not only relational and contextual expertise but also clinical facts (ICN, 2023).

## Workforce conditions, safety, and the sustainability of care

The ability to deliver safe, relational care depends fundamentally on the conditions under which nurses work (ICN, 2024). Several contributions in this issue confront this reality directly.

Workplace violence emerges as a serious occupational hazard with profound psychological consequences. Evidence linking exposure to violence with post-traumatic stress symptoms among nurses reinforces what many already know: violence is not an unfortunate anomaly but a predictable risk in under-resourced, high-pressure environments (Graske et al., 2025). Crucially, the availability of employer-provided support significantly reduces the likelihood of severe psychological harm, underscoring that organisational response matters as much as incident prevention (Christensen 2025; Graske et al., 2025).

Staffing levels and workforce composition are also examined, albeit with more ambiguous findings. The absence of a clear relationship between staffing numbers, years of service or nationality diversity, with patient satisfaction challenges simplistic assumptions (Placido and Yahsaneh, 2025; Ramdani 2025). Rather than undermining the importance of staffing, these findings point to the limits of satisfaction metrics and the need to look beyond numbers to understand care quality (WHO, 2025).

Workforce sustainability is further complicated by chronic fatigue, emotional labour, and the accumulation of unresolved stress. Qualitative insights into sleep disruption among people with restless legs syndrome indirectly echo this concern (Berdida 2025; Odzakovic, 2025).

## From data to meaning: judgement, technology and the future of nursing

The final theme in this issue looks forward, asking how nursing judgement can be sustained in increasingly data-driven healthcare systems (Alves and Alves, 2025). Framing clinical judgement as practical wisdom highlights what is at stake. Nursing judgement is not merely calculation; it integrates evidence, experience, ethical reasoning, and relational understanding (Benner, 1984; Tanner, 2006). When technologies present recommendations without transparency or invite uncritical reliance, they threaten to erode professional agency and the development of expertise (Alves and Alves, 2025). The proposed stance of epistemic heed offers a pragmatic way forward. When paired with reflective supervision, education, and supportive systems, technology may enhance rather than undermine nursing practice.

## Conclusion: sustaining nursing’s capacity to care

Across diverse settings and methods, the contributions in this issue converge on a shared message: nursing’s value lies in its ability to transform complexity into safe, ethical, and meaningful care. Planned education, prevention, continuity, shared decision-making, and reflective judgement are not optional extras.

At a time when international bodies such as the ICN continue to warn of escalating risks to the nursing workforce, this issue offers grounded evidence and thoughtful critique. Sustaining nursing’s human work is no longer simply a professional concern; it is a prerequisite for safe and just healthcare.
